# Supplementary material for: Predicting visual function by interpreting a neuronal wiring diagram
Source: Nature. 2024 Oct 2;634(8032):113–23. doi: 10.1038/s41586-024-07953-5 (PMC11446822; doi:10.1038/s41586-024-07953-5)
Supplement: Supplementary file 6 — CRF and ERF predictions for individual TmY4 and TmY9 cells. Analogous to Supplementary Data 3, but for TmY target types. Shown are the top four monosynaptic pathways, the strongest pathway passing through each of the top ten intermediary types (ranking from Extended Data Fig. 7), and the trisynaptic pathway Tm1–TmY–Dm3–TmY (see the section entitled Prediction of spatial normalization). [file 41586_2024_7953_MOESM6_ESM.zip › DataS4/TmY9q/720575940618119483.pdf]

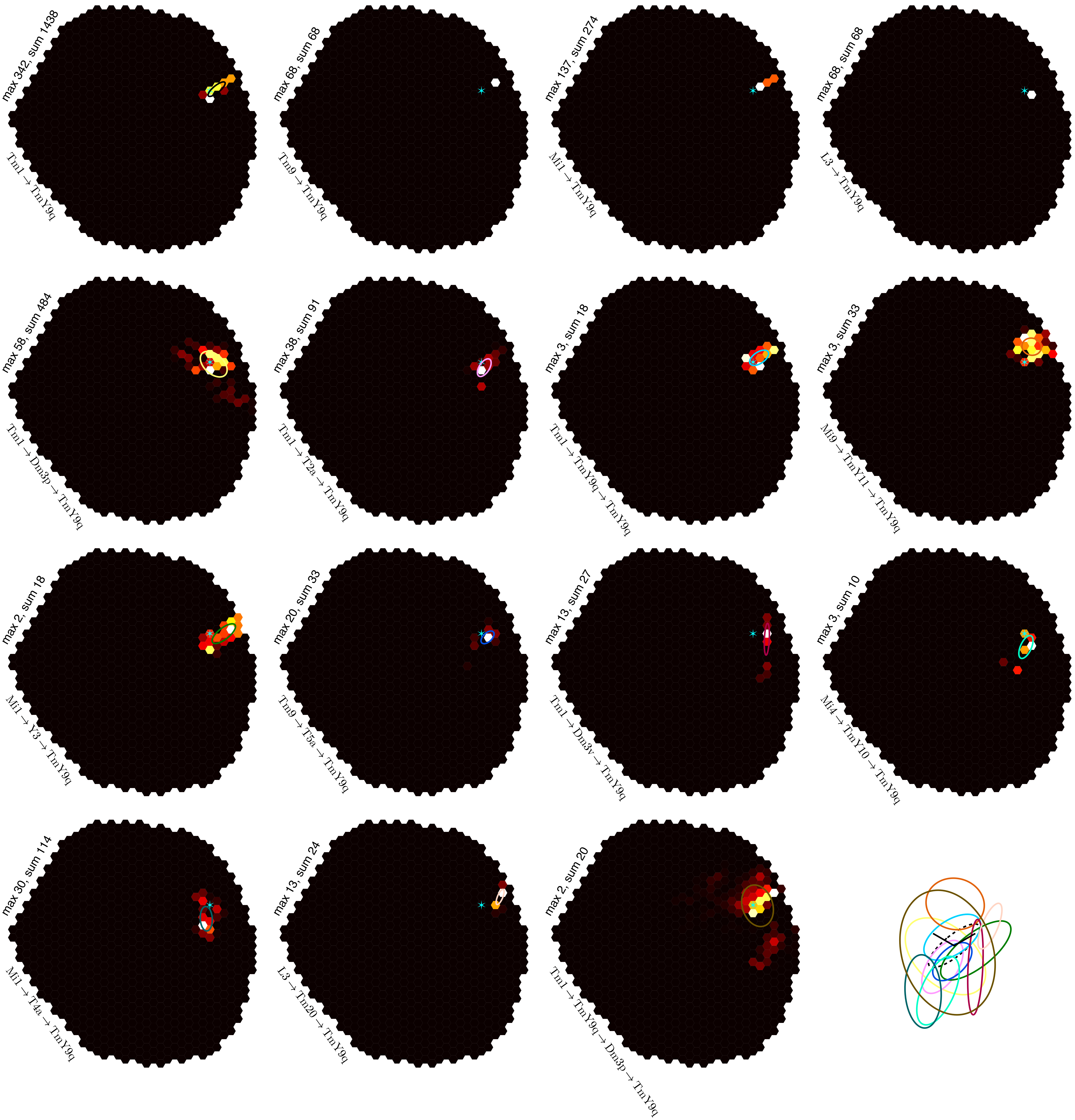
max 342, sum 1438
Tm1 → TmY9q
max 68, sum 68
Tm9 → TmY9q
max 137, sum 274
Mi1 → TmY9q
max 68, sum 68
L3 → TmY9q
max 58, sum 484
Tm1 → Dm3p → TmY9q
max 38, sum 91
Tm1 → T2a → TmY9q
max 3, sum 18
Tm1 → TmY9q → TmY9q
max 3, sum 33
Mi9 → TmY11 → TmY9q
max 2, sum 18
Mi1 → Y3 → TmY9q
max 20, sum 33
Tm9 → T5a → TmY9q
max 13, sum 27
Tm1 → Dm3v → TmY9q
max 3, sum 10
Mi4 → TmY10 → TmY9q
max 30, sum 114
Mi1 → T4a → TmY9q
max 13, sum 24
L3 → Tm20 → TmY9q
max 2, sum 20
Tm1 → TmY9q → Dm3p → TmY9q
